# The Role of Azacitidine in the Treatment of Elderly Patients with Acute Myeloid Leukemia: Results of a Retrospective Multicenter Study

**DOI:** 10.4274/tjh.2015.0203

**Published:** 2016-12-01

**Authors:** Anıl Tombak, Mehmet Ali Uçar, Aydan Akdeniz, Eyüp Naci Tiftik, Deniz Gören Şahin, Olga Meltem Akay, Murat Yıldırım, Oral Nevruz, Cem Kis, Emel Gürkan, Şerife Medeni Solmaz, Mehmet Ali Özcan, Rahşan Yıldırım, İlhami Berber, Mehmet Ali Erkurt, Tülin Fıratlı Tuğlular, Pınar Tarkun, İrfan Yavaşoğlu, Mehmet Hilmi Doğu, İsmail Sarı, Mustafa Merter, Muhit Özcan, Esra Yıldızhan, Leylagül Kaynar, Özgür Mehtap, Ayşe Uysal, Fahri Şahin, Ozan Salim, Mehmet Ali Sungur

**Affiliations:** 1 Mersin University Faculty of Medicine, Department of Hematology, Mersin, Turkey; 2 Osmangazi University Faculty of Medicine, Department of Hematology, Eskişehir, Turkey; 3 Gülhane Training and Research Hospital, Clinic of Hematology, Ankara, Turkey; 4 Çukurova University Faculty of Medicine, Department of Hematology, Adana, Turkey; 5 Dokuz Eylül University Faculty of Medicine, Department of Hematology, İzmir, Turkey; 6 Atatürk University Faculty of Medicine, Department of Hematology, Erzurum, Turkey; 7 İnönü University Faculty of Medicine, Department of Hematology, Malatya, Turkey; 8 Marmara University Faculty of Medicine, Department of Hematology, İstanbul, Turkey; 9 Kocaeli University Faculty of Medicine, Department of Hematology, Kocaeli, Turkey; 10 Adnan Menderes University Faculty of Medicine, Department of Hematology, Aydın, Turkey; 11 Pamukkale University Faculty of Medicine, Department of Hematology, Denizli, Turkey; 12 Ankara University Faculty of Medicine, Department of Hematology, Ankara, Turkey; 13 Erciyes University Faculty of Medicine, Department of Hematology, Kayseri, Turkey; 14 Ege University Faculty of Medicine, Department of Hematology, İzmir, Turkey; 15 Akdeniz University Faculty of Medicine, Department of Hematology, Antalya, Turkey; 16 Düzce University Faculty of Medicine, Department of Biostatistics, Düzce, Turkey

**Keywords:** Azacitidine, Acute myeloid leukemia, Elderly, Bone marrow blasts, Prognostic factors, overall survival

## Abstract

**Objective:**

In this study, we aimed to investigate the efficacy and safety of azacitidine (AZA) in elderly patients with acute myeloid leukemia (AML), including patients with >30% bone marrow (BM) blasts.

**Materials and Methods:**

In this retrospective multicenter study, 130 patients of ≥60 years o ld who were ineligible for intensive chemotherapy or had progressed despite conventional treatment were included.

**Results:**

The median age was 73 years and 61.5% of patients had >30% BM blasts. Patients received AZA for a median of four cycles (range: 1-21). Initial overall response [including complete remission (CR)/CR with incomplete recovery/partial remission] was 36.2%. Hematologic improvement (HI) of any kind was documented in 37.7% of all patients. HI was also documented in 27.1% of patients who were unresponsive to treatment. Median overall survival (OS) was 18 months for responders and 12 months for nonresponders (p=0.005). In the unresponsive patient group, any HI improved OS compared to patients without any HI (median OS was 14 months versus 10 months, p=0.068). Eastern Cooperative Oncology Group performance status of <2, increasing number of AZA cycles (≥5 courses), and any HI predicted better OS. Age, AML type, and BM blast percentage had no impact.

**Conclusion:**

We conclude that AZA is effective and well tolerated in elderly comorbid AML patients, irrespective of BM blast count, and HI should be considered a sufficient response to continue treatment with AZA.

## Abstract (tr)

**Amaç:**

Bu çalışmada, kemik iliğindeki (Kİ) blast oranı >%30 olan olguları da içeren akut miyeloid lösemili (AML) yaşlı hastalarda, azasitidinin (AZA) etkinliğinin ve güvenliğinin araştırılmasını amaçladık.

**Gereç ve Yöntemler:**

Bu geriye dönük, çok merkezli çalışmaya, yoğun kemoterapi için uygun olmayan ya da konvansiyonel tedavilere rağmen hastalığı ilerleyen ≥60 yaştaki 130 hasta dahil edildi.

**Bulgular:**

Ortanca yaş 73 idi, hastaların %61,5’inde Kİ blast oranı >%30 olarak bulundu. Hastalar, ortanca 4 döngü (1-21 aralığında) AZA almıştı. Başlangıç genel yanıt oranı [tam yanıtı (TY)/eksik düzelmenin olduğu TY/kısmi yanıtı içeren] %36,2 idi. Herhangi bir hematolojik düzelme (HD), tüm hastaların %36,2’sinde tespit edildi. HD tedaviye yanıtsız hastaların %27,1’inde de saptandı. Ortanca genel sağkalım, yanıt verenlerde 18 ay, yanıt vermeyenlerde 12 ay idi (p=0,005). Tedaviye yanıtsız hasta grubunda HD’nin, HD olmayanlara kıyasla genel sağkalımı arttırdığı görüldü (ortanca sağkalım 14 aya kıyasla 10 ay, p=0,068). Doğu Kooperatif Onkoloji Grubu performans durumunun <2 olması, AZA döngü sayısının artması (≥5 döngü) ve herhangi bir HD olması, daha iyi genel sağkalımı öngörüyordu. Yaşın, AML tipinin, Kİ blast yüzdesinin etkisi yoktu.

**Sonuç:**

AZA, yaşlı, eşlik eden hastalıkları olan AML’li hastalarda, Kİ blast sayısından bağımsız olarak etkindir ve iyi tolere edilmektedir ve HD’nin, AZA ile tedaviye devam etmek için yeterli bir yanıt olduğu göz önünde bulundurulmalıdır.

## INTRODUCTION

Acute myeloid leukemia (AML) is predominantly a disease of older patients with a median age at diagnosis of ~70 years [1,2]. Older patients with AML have significant comorbidities, a poorer performance status, more unfavorable cytogenetic abnormalities, and a higher incidence of secondary AML than their younger counterparts and only approximately 1/3 of elderly AML patients are eligible for conventional anthracycline/cytarabine-based intensive chemotherapeutic approaches [3,4,5]. However, overall results of intensive chemotherapy remain poor even for those who do meet inclusion criteria for such treatment [1,3,4,5]. Patients not suitable for intensive chemotherapy or who did not respond to these treatment options are frequently offered best supportive care (BSC) only, and the prognosis is dismal [6,7].

The hypomethylating agents decitabine and azacitidine (AZA) have significant activity in patients with a myelodysplastic syndrome (MDS) [8,9]. The use of AZA was associated with improved survival when compared to BSC or low-dose cytarabine in patients with high-risk MDS, including those with marrow blast counts ranging from 20% to 30%, leading to AZA approval in these disease categories [8,10]. In untreated or relapsed/refractory AML, a few studies have also shown significant response rates of AZA therapy [11,12,13,14,15]. However, there are limited data showing the efficacy of AZA in AML patients with >30% bone marrow (BM) blasts.

In this retrospective multicenter study, we aimed to investigate the efficacy and safety of AZA in elderly patients with AML (including patients with >30% BM blasts) defined according to the World Health Organization (WHO).

## MATERIALS AND METHODS

### Patients and Eligibility Criteria

Between June 2009 and June 2014, 130 patients of ≥60 years old with AML from 16 specialized centers for hematology in Turkey, defined according to WHO criteria, were included. Eligibility criteria included all ≥60-year-old AML patients who were treated with at least one dose of AZA. Demographic data, comorbidities (cardiovascular diseases, diabetes mellitus, prior/concomitant malignancies, pulmonary disease, renal insufficiency), Eastern Cooperative Oncology Group (ECOG) status, transfusion dependency, cytogenetic risk status according to the refined Medical Research Council (MRC) criteria [16], treatment prior to AZA, and concomitant treatments were recorded. AZA was administered at 75 mg/m^2^ subcutaneously daily for 7 days and 100 mg/m^2^ subcutaneously daily for 7 days. The local ethics committee approved this retrospective analysis.

### Efficacy and Safety Assessments

Assessment of response was performed after a median of 4 cycles of AZA. BM aspirations/punctures were performed and reviewed by the principal investigator (hematologist) at each center. Overall responses including complete remission (CR), partial remission (PR), CR with incomplete recovery (CRi), and failure were defined according to International Working Group (IWG) criteria for AML [17]. Patients with persisting peripheral blasts following AZA were also classified as nonresponders if BM puncture was not performed. Hematologic improvement (HI) was evaluated using IWG criteria for MDS from the collected transfusion records of the patients [18]. Specific hematologic and nonhematological adverse events were graded according to Common Terminology Criteria for Adverse Events (CTCAE) v4.0, published on 28 May 2009, by the National Cancer Institute. All data including response, HI, and adverse events were determined and recorded by principal hematologists at the respective centers.

### Statistical Analysis

Categorical data were analyzed by chi-square or Fisher’s exact test according to expected count rule and summarized as frequency and percentage. Both univariate and multivariate logistic regression analyses were used to obtain the odds ratio (OR) of variables that significantly affected response rate. Survival times and curves were estimated by Kaplan-Meier method and compared by log-rank test. Both univariate and multivariate Cox regression models were constructed for obtaining the hazard ratio (HR) of variables that significantly affected survival. Statistical analyses were performed with PASW v.18 software (Predictive Analytics Software is a registered trademark of SPSS Inc.), and p<0.05 was considered statistically significant.

## RESULTS

### Patient Characteristics

Patient baseline characteristics are summarized in Table 1. A total of 130 patients with AML (58 women, 72 men) receiving AZA were included in the study. Median age was 73, ranging from 60 to 88 years; 31.5% (n=41) of patients were 60-69 years old, 49.2% (n=64) were 70-79 years old, and 19.2% (n=25) were ≥80 years old. ECOG performance status (ECOG-PS) was ≥2 in 54.6% (n=71) and there were comorbidities in 66.2% (n=86) of the cases; of these, 89.5% (n=77) had <3 and 10.5% (n=9) had ≥3 comorbidities. Lactate dehydrogenase (LDH) level was <225 IU/L in 20.8% (n=27) and was ≥225 IU/L in 75.4% (n=98) of the cases, and 40.8% (n=53) of the patients had a leukocyte count of >10x10^9^/L. Median absolute neutrophil count (ANC) was 1.1x10^9^/L, median hemoglobin concentration was 8.7 g/L, and median platelet count was 57x10^9^/L. Ninety-four (72.3%) patients had peripheral blood blasts and 80 patients (61.5%) had >30% BM blasts. One hundred and twelve patients (86.2%) required erythrocyte and/or thrombocyte transfusion (transfusion-dependent), while 5.4% had an unfavorable karyotype and 50.8% had an intermediate karyotype according to MRC criteria [16].

### Treatment Modalities

While 54.6% (n=71) of the patients did not receive any treatment prior to AZA, intensive chemotherapy, hydroxyurea, low-dose cytarabine, erythropoietin-stimulating agents, iron chelation therapy, lenalidomide, and granulocyte-colony stimulating factor (G-CSF) were used in 16.9% (n=22), 16.9% (n=22), 5.4% (n=7), 3.1% (n=4), 1.5% (n=2), 0.8% (n=1), and 0.8% (n=1) of the cases, respectively.

AZA was administered as first-line therapy in 79.2% of patients (n=103). No CR or early relapse after conventional (intensive) chemotherapy and after other disease-modifying treatments was the reason for AZA treatment in 13.8% (n=18) and 6.9% (n=9) of patients, respectively. AZA was administered at 75 mg/m2 subcutaneously daily for 7 days and 100 mg/m2 subcutaneously daily for 7 days in 81.5% and 18.5% of the patients, respectively. A median number of 4 (range: 1-21) AZA courses were given in 28-day intervals. In all AZA cycles, hydroxyurea (11.5%) or G-CSF (7.7%) was given concomitantly when deemed necessary by the treating physician.

### Response to Azacitidine and Survival

Initial overall response (including CR/CRi/PR according to IWG) was evaluated after a median of 4 cycles of AZA. While there was no response in 53.8% (n=70) of patients, CR, CRi, and PR were documented in 13.1% (n=17), 6.2% (n=8), and 16.9% (n=22) of the cases, respectively (Table 2). Any HI according to IWG criteria was documented in 37.7% (n=49) of the patients; neutrophil, erythroid, and platelet responses were observed in 18.5% (n=24), 3.8% (n=5), and 15.4% (n=20) of the patients, respectively (Table 2). HI was also documented in 27.1% (n=19) of 70 patients who were unresponsive to treatment.

Median overall survival (OS) was 12.3 [95% confidence interval (CI): 10.1-14.6] months as of the first diagnosis of AML. Disease-free survival (DFS) and event-free survival (EFS) were 16.2 (95% CI: 6.7-25.7) and 8.3 (95% CI: 6.1-10.6) months, respectively. Median OS was 18 (95% CI: 10.6-25.4) months for responders (defined as CR/CRi/PR) and 12 (95% CI: 9.2-14.8) months for nonresponders (p=0.005). In addition, median OS was 14 (95% CI: 4.1-23.9) months in patients unresponsive to treatment (without CR/CRi/PR) but with any HI (n=19), and was 10 (95% CI: 4.1-15.9) months in patients unresponsive to treatment and also without any HI (n=51) (p=0.068). Median OS of the patients who received AZA as a rescue after intensive chemotherapy was 24 (95% CI: 13.3-34.7) months as of the first diagnosis of AML.

In univariate analysis the following parameters had a significant effect on both treatment response and OS: ECOG-PS score, number of AZA cycles, and any HI. However, sex, age, absolute number of comorbidities, presence of peripheral blasts, AML type, leukocyte count at the time of diagnosis, treatment prior to AZA, and BM blast count had no significant impact on treatment response and OS (Table 3). Since the number of patients with good (n=1) and poor-risk cytogenetics (n=7) was low, the effect of cytogenetics on response to treatment and OS was not evaluated. Similarly, since the number of patients receiving AZA at 100 mg/m2 was low (n=24), the impact of altered dosing schedules of AZA was not evaluated.

In multivariate analysis, all variables with p<0.05 in univariate analysis were included, and it was found that increasing number of AZA cycles (≥5) was associated with a better response rate and ECOG-PS score of ≥2 was a significant predictor of shorter OS (Table 4).

### Toxicity and Adverse Events

A total of 351 adverse events were documented. CTCAE grade 3-4 neutropenia, thrombocytopenia, and anemia were documented in 34.6%, 40.8%, and 39.2% of patients, respectively. Febrile neutropenia was documented in 60.8% of the patients. Other nonhematological toxicities were usually mild, the most common adverse events being mucositis, diarrhea, injection site pain, and nausea.

## DISCUSSION

Incidence of AML increases with age and most patients are deemed unsuitable for intensive treatment options. Outcomes following conventional chemotherapeutic approaches are poor. AZA is a hypomethylating agent, and owing to its acceptable tolerability profiles and emerging evidence of clinical efficacy, it may provide an exciting approach to the treatment of elderly patients with AML. It is licensed for patients with 20%-30% blasts and it confers a survival benefit in these patients [14]; studies suggest 10%-20% CR rates with AZA [14,19,20,21] and these patients have OS rates equivalent or superior to other conventional treatments [14,19,21]. However, data on AZA activity in AML patients with BM blast counts of >30% are limited and the drug can be used off-label in these patients, although several analyses have also suggested that AZA is active and well tolerated in patients with >30% BM blasts as well [11,12,15,20].

In the current study, we retrospectively analyzed the efficacy and toxicity of AZA in 130 patients with AML who were ≥60 years of age, and this cohort also included 80 patients (61.5% of the cases) with >30% BM blasts. We found a CR rate similar to the CR rates of recent studies [14,19,20,21], which was documented in 13.1% of our patient cohort. Median OS was 12.3 months and OS was longer in responders compared to nonresponders. We also showed that AZA was effective in the group with >30% BM blasts and that BM blast count of 20%-30% versus >30% has no significant impact on response rate or OS. In addition, although the response rate and OS were somewhat poor with the presence of peripheral blasts, these results were not statistically significant. In a study conducted by van der Helm et al. it was shown that BM blast percentage had no impact on OS as well [22]. In a recent phase 3 study of AZA versus conventional care regimens in newly diagnosed AML patients of ≥65 years with >30% BM blasts, Dombret et al. confirmed the clinical observation that AZA can have meaningful clinical activity (e.g., transfusion independency) and improve survival, even though no CR is achieved [23]. Thus, we recommend that AML patients with >30% BM blasts should not be precluded from treatment with AZA and the presence of peripheral blasts should not be a reason for therapy cessation.

HI was found to be a predictor of prolonged survival; significantly longer OS was observed in patients achieving any kind of HI compared to patients without any HI (p=0.002), and similar results have been shown in recent AML patient cohorts [15,20]. However, interestingly, we also found that in the unresponsive (without CR/CRi/PR) patient group, OS was significantly longer for patients who achieved HI compared to those without any HI (p=0.068). In other words, although this was not a statistically significant result, HI without CR/CRi or PR was also associated with a better OS. If commonly used AML response criteria were to be applied [17], patients who experience HI without CR, CRi, or PR would be called nonresponders and treatment with AZA would be discontinued. With these results, we can conclude that, since cytopenias are the cause of mortality in the majority of patients with AML, the goal of therapy with AZA should not just be CR or PR, and therapy should be continued in patients with any HI although there is not any simultaneous BM response.

Another result of our study was that, as the number of AZA courses increased, response rate and OS increased. This is not a surprise, because the epigenetic therapeutic effects of AZA are dependent on the S-phase of the cell cycle and each cycle of therapy can only affect the fraction of the malignant clone that enters the S-phase. Thus, the best responses can occur after as many as 12 cycles of therapy, with a median of 3-3.5 cycles [24]. Therefore, the treatment should not be interrupted in the early stages of therapy and it should be continued as long as the response is durable and/or until overt clinical progression occurs.

We confirm the results of previous studies [15,20,25] that WHO-AML type, treatment prior to AZA, sex, and age had no significant effect on OS. Not the age but rather the absolute number of comorbidities may adversely affect OS. In our study, a cut-off of <3/≥3 comorbidities was analyzed and there was a trend for reduced OS for patients with ≥3 comorbidities, which was, however, not statistically significant (p=0.662). Similarly, LDH of ≥225 IU/L was associated with reduced OS (p=0.018), but it had no impact on treatment response (p=0.758). Importantly, ECOG-PS of ≥2 was found to be the only baseline factor affecting OS in both univariate and multivariate analysis. Recently, an Austrian group reported that the absolute number of comorbidities and LDH of ≥225 IU/L were independent adverse predictors of OS in their larger cohort (n=302) [15] and borderline significant in their previously published smaller cohort (n=155) [20]. As we found in our study, ECOG-PS of ≥2 was an independent adverse predictor of OS in both of the Austrian studies [15,20], and in a French study as well [25]. In our opinion, older age, WHO-AML type, prior treatments, and LDH level should not lead to a decision to withhold treatment of AZA in favor of BSC if the patient has an ECOG-PS score of <2.

Elevated leukocyte count had no impact on OS in our study, but conflicting results exist in the literature. Both aforementioned Austrian publications showed that leukocyte count of neither >10x10^9^/L nor >15x10^9^/L significantly affected OS [15,20], but the French publication showed a significant effect of leukocyte count of >15x10^9^/L on OS [25]. We think that AML patients with high leukocyte counts should not be precluded from treatment with AZA, and cytoreduction with hydroxyurea or low-dose cytarabine may be an appropriate approach in such patients.

As expected, transfusion dependence prior to AZA was associated with reduced OS in our study in univariate analysis, which was, however, not statistically significant (p=0.077). Transfusion dependence was not a predictor of reduced OS in the multivariate analysis of the Austrian studies, as well [15,20].

The most commonly observed toxicity was febrile neutropenia, at a rate higher than seen in the literature [12,13,15]. Other nonhematological toxicities were mild. However, due to the retrospective nature of this analysis, toxicities in general were probably underestimated.

Certainly, our study has several shortcomings, since it was a retrospective study, the patient population was heterogeneous, and the effect of cytogenetics on response to treatment was not evaluated.

In conclusion, AZA is effective and well tolerated in elderly comorbid AML patients with fewer required erythrocyte and platelet transfusions, irrespective of BM blast count. HI should be considered a sufficient response to continue treatment with AZA and treatment should not be interrupted since OS and response to treatment increase with increasing numbers of AZA cycles.

## Ethics

Ethics Committee Approval: This study was approved by Mersin University Ethics Committee, Informed Consent: It is a retrospective study.

## Figures and Tables

**Table 1:**
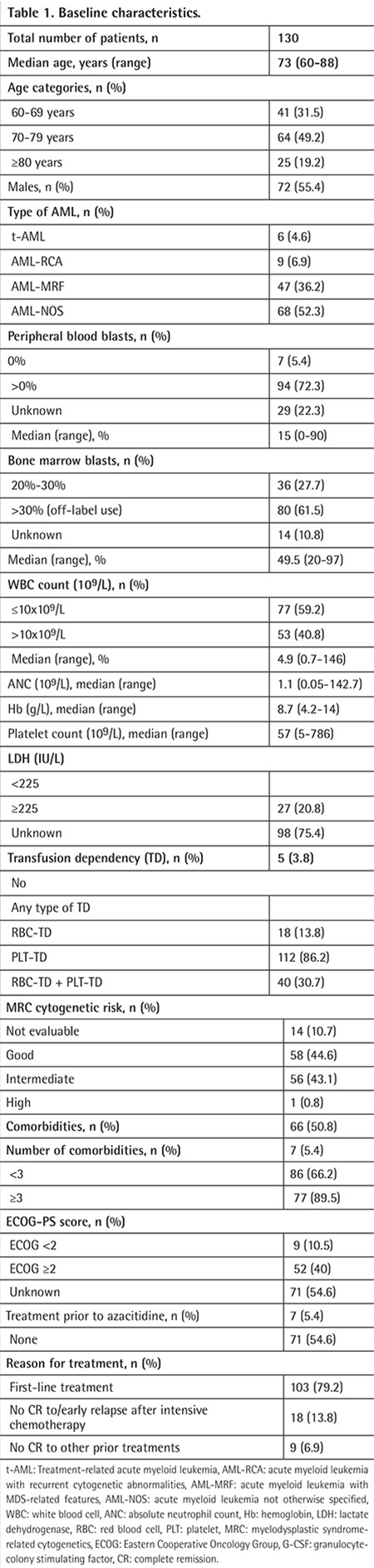
Baseline characteristics.

**Table 2:**
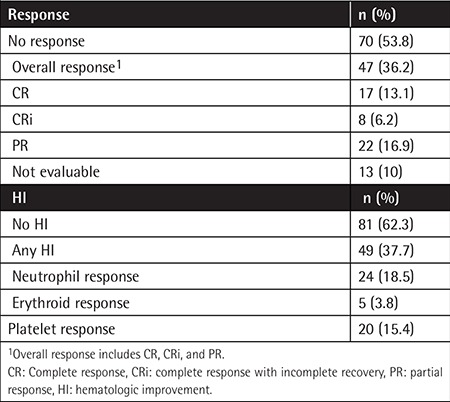
Response to azacitidine according to International Working Group criteria.

**Table 3:**
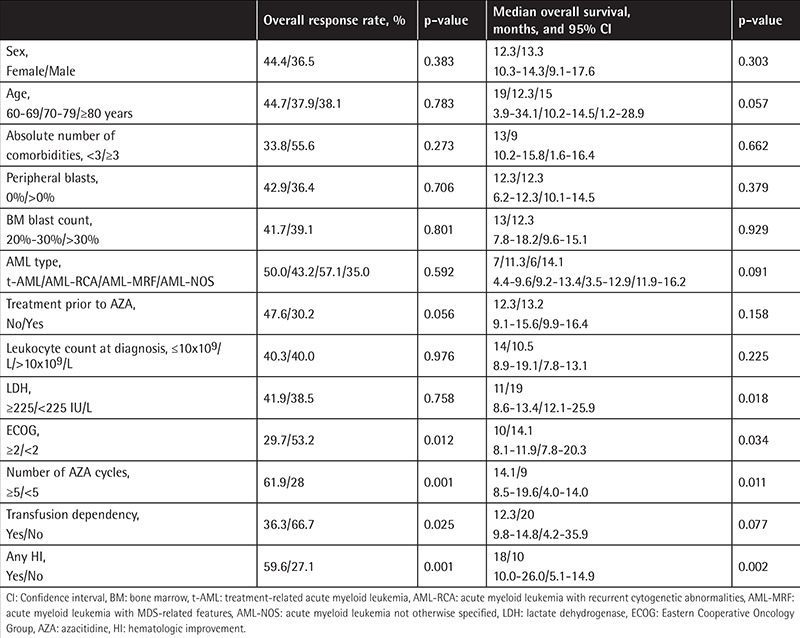
Univariate analysis for response and overall survival.

**Table 4:**
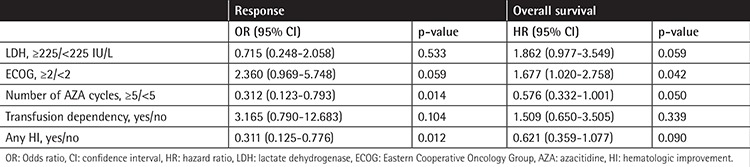
Multivariate analysis for response and overall survival.
